# Synthetic 9-*cis*-beta-carotene inhibits photoreceptor degeneration in cultures of eye cups from rpe65rd12 mouse model of retinoid cycle defect

**DOI:** 10.1038/s41598-018-24439-3

**Published:** 2018-04-17

**Authors:** Ifat Sher, Adi Tzameret, Sara Peri-Chen, Victoria Edelshtain, Michael Ioffe, Alon Sayer, Ludmila Buzhansky, Ehud Gazit, Ygal Rotenstreich

**Affiliations:** 1 Goldschleger Eye Institute, Sheba Medical Center, Tel-Hashomer, Israel; 2 Sackler Faculty of Medicine, Tel Aviv University, Tel Aviv, Israel; 3 Department of Molecular Microbiology and Biotechnology, George S. Wise Faculty of Life Sciences, Tel Aviv University, Tel Aviv, Israel; 4 Department of Materials Science and Engineering, Iby and Aladar Fleischman Faculty of Engineering, Tel Aviv University, Tel Aviv, Israel; 5 BLAVATNIK CENTER for Drug Discovery, Tel Aviv University, Tel Aviv, Israel

## Abstract

The retinoid cycle enzymes regenerate the visual chromophore 11-cis retinal to enable vision. Mutations in the genes encoding the proteins of the retinoid cycle are the leading cause for recessively inherited retinal dystrophies such as retinitis pigmentosa, Leber congenital amaurosis, congenital cone-rod dystrophy and fundus albipunctatus. Currently there is no treatment for these blinding diseases. In previous studies we demonstrated that oral treatment with the 9-*cis*-β-carotene rich *Dunaliella Bardawil* algae powder significantly improved visual and retinal functions in patients with retinitis pigmentosa and fundus albipunctatus. Here we developed a convenient and economical synthetic route for biologically active 9-*cis*-β-carotene from inexpensive building materials and demonstrated that the molecule is stable for at least one month. Synthetic 9-*cis*-β-carotene rescued cone photoreceptors from degeneration in eye cup cultures of mice with a retinoid cycle genetic defect. This study suggests that synthetic 9-*cis*-β-carotene may serve as an effective treatment for retinal dystrophies involving the retinoid cycle.

## Introduction

The retinoid cycle is the enzyme pathway that regenerates the vision chromophore 11-*cis* retinal. Mutations in the genes encoding the retinoid binding proteins and enzymes of this pathway lead to incurable vision impairment and blinding diseases including retinitis pigmentosa (RP), Leber congenital amaurosis (LCA), congenital cone-rod dystrophy and fundus albipunctatus^1^. Among these are mutations in the genes encoding the isomerase retinal pigment epithelium-specific protein 65 kDa (RPE65) and the lecithin:retinol acyltransferase (LRAT) retinal cycle enzymes that lead to LCA and RP^2,3^.

Studies in animal models of mutations in LRAT and RPE65 suggested that 9-*cis*-retinal can replace the missing native 11-cis chromophore and efficiently bind rhodopsin, forming a light sensitive isorhodopsin *in vivo*^4–7^. Furthermore, oral treatment with 9-*cis* -retinyl acetate improved retinal function and vision in LCA patients with mutations in LRAT and RPE65^8,9^, suggesting that precursors of 9-*cis* retinoids may present a new treatment strategy for patients with mutations in the genes of the retinoid cycle.

9-*cis*-β-carotene (9CBC) is a natural source for 9 cis retinal. It can be cleaved by beta-carotene oxygenase 1 (BCMO1) to produce 9-*cis*-retinal *in vivo*^10,11^. BCMO1 is expressed in the eye in RPE cells, where it may provide the retina with supplement of retinal from dietary beta carotenes^12^. In previous studies we demonstrated that oral treatment with a powder of the 9-*cis*-β-carotene (9CBC)-rich alga *Dunaliella Bardawil* restored retinal function in patients with congenital stationary night blindness (fundus albipunctatus)^13^. In a larger clinical study that included 29 non-genetically defined RP patients, oral treatment with the 9CBC-rich alga significantly increased the retinal function in one third of the patients^14^. Since 9CBC is a known precursor of retinal^15^, we hypothesized that the therapeutic effects of 9CBC may have been mediated via production of 9-*cis*-retinal *in vivo*. No adverse effects were observed in these studies, suggesting that 9CBC may potentially be used for treatment of retinal dystrophies. Furthermore, preclinical and clinical studies suggest that 9CBC may be used for treatment of other diseases. Thus, oral treatment with the 9CBC-rich alga reduced the severity of chronic plaque psoriasis in a recent clinical trial and inhibited atherogenesis and fatty liver formation in LDL knockout mice^16,17^. In spite of the importance of 9CBC the current experimental therapy is solely based on natural alga products. In order to allow a therapeutic use of the compound there is a need for controlled production of the compound under industrial pharmaceutical settings. Challenges are related not only to the production of the compound but also for its formulation and stabilization.

Here we developed a synthetic route for 9CBC from readily available low cost materials and demonstrated that synthetic 9CBC can rescue retinal photoreceptor cells from degeneration in eye cup cultures from RPE65rd12 mouse model, that lacks the expression of the retinoid cycle RPE65 enzyme. The stability of 9CBC throughout synthesis, purification, storage and packing is vital for preserving its biological activity. In the present study, a challenging matter was ensuring the stability of the 9CBC by development of suitable preserving formulation. A number of experiments shown that oxidation was the main reason causing 9CBC degradation, especially in its pure form (the crude material was significantly less affected by oxidation). Initial stability tests indicated that synthetic 9CBC is sensitive to both oxygen and UV degradation due to the highly conjugated nature of the carbon chain, resulting in full decomposition in less than 24 hours. Attempt to stabilize 9CBC by the addition of antioxidant(s), as suggested in the literature^18^ were unsuccessful, as such a composition has quite rapidly degraded. As has been surprisingly found and shown herein, a 9CBC formulation containing, as non-active ingredients, a viscosity enhancing agent such as a lecithin, e.g., a soybean lecithin, a medium chain triglyceride such as miglyol, or a polysorbate such as polysorbate 20 and polysorbate 40 as antioxidants, are highly stable for a long period of time. In a further aspect, the present formulation thus provides a pharmaceutical composition, comprising an active agent selected from 9CBC or a derivative thereof of the formula C as defined above, a thickening- or solidifying-, i.e., viscosity enhancing, agent, and an antioxidant. Such pharmaceutical formulations are useful for treatment of any biological indication in which administration of 9CBC is useful, e.g., neuro-retinal degeneration.

## Results

### 9-*cis*-β-Carotene synthesis

**Figure 1:**
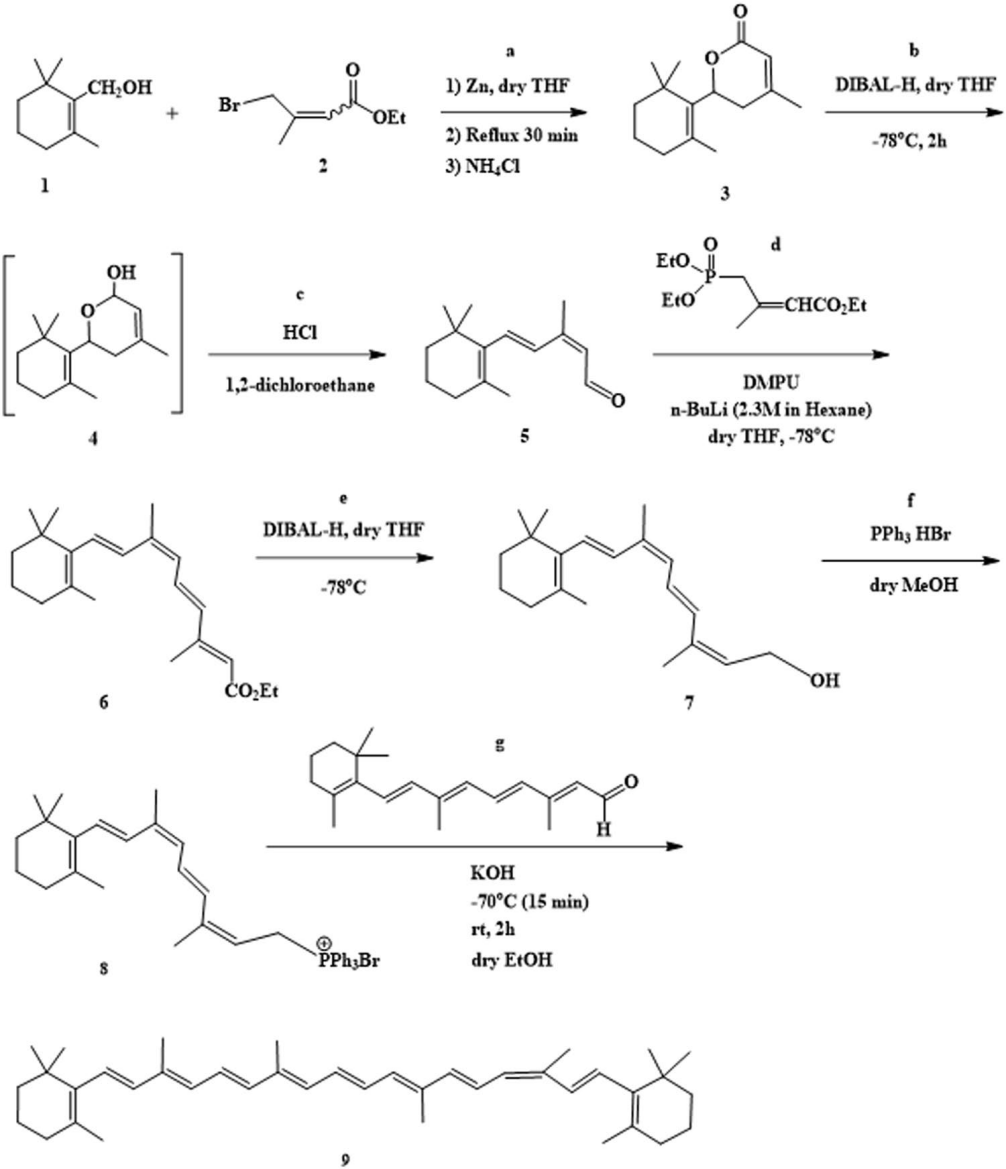
Synthesis of 9-cis-β-carotene.

**Figure 2:**
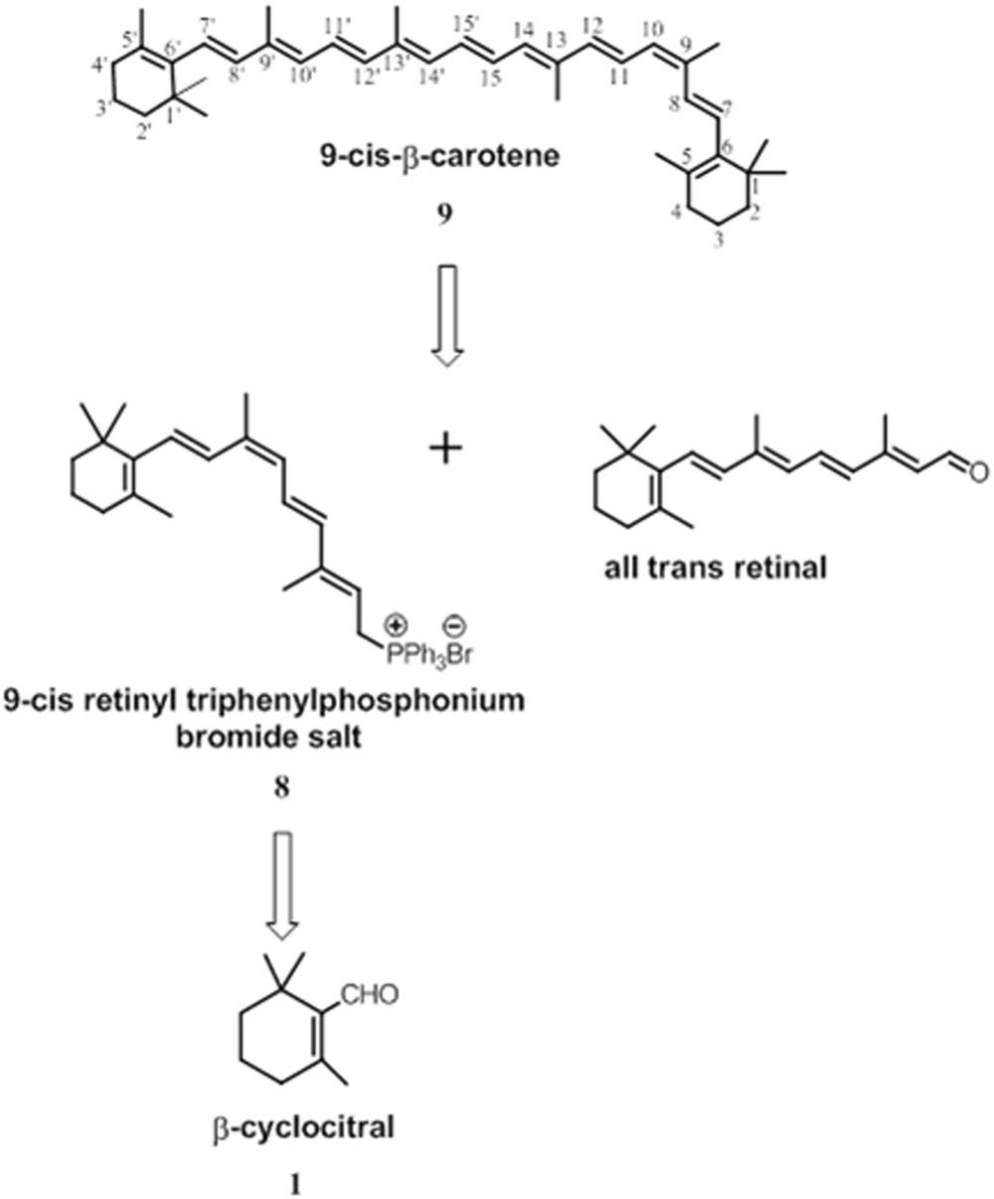
Synthesis of ethyl 4-bromo-3-methylbut-2-enoate.

As described and depicted in Fig. 1, 9-*cis*-β-carotene (9CBC) was obtained by multi-step synthesis (8 steps). The building blocks for the synthesis of 9CBC were β-cyclocitral and all-*trans* retinal. 9CBC was afforded by Wittig reaction of all-*trans* retinal and 9-*cis*-retinyl triphenylphosphonium bromide salt^19^ which was obtained from the building block, β-cyclocitral (Fig. 2).

### Synthesis of ethyl 4-bromo-3-methylbut-2-enoate

As depicted in Fig. 2, N-bromosuccinimide (85 mmol 15.1 g) and a catalytic amount of AIBN (0.02 g) were added to a solution of ethyl 3,3-dimethylacrylate (78 mmol, 10 g) in carbon tetrachloride (128 mL) and the mixture was heated under reflux for 3 h. It was then cooled and filtered, and the precipitate was washed with chloroform. The combined organic phases were washed with saturated aqueous sodium sulfite and brine, dried with (MgSO_4_), and concentrated under reduced pressure to give the (E) and (Z)−4-bromo-3-methylbut-2-enoate. 4-Bromo-3-methylbut-2-enoate was distilled out under vacuum (3.6 mbar, 84–86 °C). HPLC analysis was done using LiChrocart column (C18-RP (5 µm)) and the following method: elution of CH_3_CN/H_2_O, 60:40 to 100:0, in 12 min (flow rate 1 mL/min). Retention time of ethyl 4-bromo-3-methylbut-2-enoate was 7 min (purity: 96%). The spectral data were consistent with literature^20^.

### Reformatsky reaction (lactone ring formation) (step a)

As depicted in Fig. 1, step a, in a 100 mL round-bottomed flask fitted with a reflux condenser, nitrogen inlet, and addition funnel, 2.5 grams of zinc dust (38.28 mmol) and 4 mL of dry tetrahydrofuran were added. The β-cyclocitral (36.45 mmol, 5.88 mL) and ethyl 4-bromo-3-methylbut-2-enoate (36.51 mmol, 5.52 mL) were added to the addition funnel with 15 mL of dry tetrahydrofuran. The reactants were added dropwise following an initial addition of 1–2 mL to initiate the reaction. The rate of addition was adjusted to maintain a gentle reflux, with ca. 20–30 min for complete addition. The reaction solution was then heated to reflux for 30 minutes. After cooling to room temperature, 15 mL of saturated ammonium chloride solution was added and the solution was stirred for 30 min. The reaction solution was worked up with an addition of another 30 mL of saturated ammonium chloride solution to a 500 mL separatory funnel and diethyl ether extraction (3 × 25 mL). The combined organic layers were washed with brine and dried over MgSO4. The organic phase was evaporated to give 8.95 g of crude product. The product was dissolved in hot hexane and allowed to recrystallize with cooling. The white solid was filtered and collected to yield 4.68 g lactone (55%). ^1^H-NMR (CDCl_3_, 400 MHz); 5.82 (s, 1 H), 4.96 (dd, 13.4, 4.4 1 H), 2.88 (dd, 13.4, 18.5, 1 H), 2.16 (dd, 18.5, 3.4, 1 H), 1.98 (s, 3 H), 1.96 (m, 1 H), 1.75 (s, 3 H), 1.57–1.65 (m, 2 H), 1.42–1.50 (m, 2 H), 1.09 (s, 3 H), 0.97 (s, 3 H). MS (ES + ) calculated for C_15_H_22_O_2_ was 234.3, found 235.3 (M-H^+^).

### Lactone reduction (step b)

As depicted in Fig. 1, step b, to 24 mL of tetrahydrofuran (THF) lactone was added (14.08 mmol, 3.3 g). The resulting solution was cooled in a −78 °C bath and with constant stirring DIBAL (18.4 mL, 1.0 M in CH_2_Cl_2_) was added dropwise during 2 hours, never allowing the solution to warm above −40 °C. After 15–20 minutes the reaction was completed, by TLC monitoring (Et_2_O/Hexane; 40:60). Five hundred ml of 10% H_2_O/THF were added to the cooled solution as a quenching solution. The solution was removed from the bath and allowed to warm to room temperature, while monitoring the rate of warming. After reaching 0°−15 °C, the solution was cooled by using of the cooling bath again. At this point, 2 g of Na_2_SO_4_ was added and the resulting suspension was stirred for 0.5 hours. The solution was filtered through a bed of celite, the bed was washed with THF and the solution was concentrated to yield oil (2.76 g). According to MS (ES+) the aldehyde (step c) was formed with a mass of 219.3 (M-H^+^).

### Ring opening of lactol (aldehyde formation) (step c)

To a solution of 2.76 g of the lactol in 23 mL of 1,2-dichloroethane 11.5 mL of 1 N HCl solution were added. The two phase solution was heated to 40 °C, and monitored by TLC. The solution turned orange after 30 min. The reaction was found to be complete after 12.5 hours (formed an orange-red solution). Then, 50 mL of saturated Na_2_CO_3_ was carefully added to the organic layer with stirring. The aqueous layer was washed with two portions of dichloromethane and the collective organic layers were washed once with brine. The organic solution was then dried over K_2_CO_3_ (anhydrous) and concentrated in vacuo to afford a red-orange oil to afford 2.274 grams (60% for steps b and c). ^1^H-NMR (CDCl_3_, 400 MHz):10.16 (d, 8.0, 1 H), 7.08 (d, 16.0, 1 H), 6.63 (d, 15.0, 1 H), 5.86 (d, 8.0, 1 H), 2.12 (s, 3 H), 2.05 (t, 6.0, 2 H), 1.75 (s, 3 H), 1.57–1.75 (m, 2 H), 1.40–1.53 (m, 2 H), 1.05 (s, 6 H). MS (ES+) calculated for C_15_H_22_O was 218.3, found 219.3 (M-H^+^). HPLC analysis was performed using LiChrocart column [C18-RP (5 µm)] and the following method: elution of CH_3_CN/H_2_O, 60:40 to 100:0, in 12 min and then 100% CH_3_CN for 5 min (flow rate 1 mL/min, λ = 396 nm). Retention time of lactone was 13 min (purity: 90%).

### Horner-Emmons reaction (9-cis retinyl ester formation)

A solution of triethyl-3-methyl-4-phosphono-2-butenoate (14.42 mmol, 3.51 mL) in anhydrous THF (21.7 mL) was cooled to 0 °C and treated with anhydrous DMPU (29.7 mmol, 3.57 mL) and *n*-BuLi in hexanes (6.08 mL of 2.5 M solution, 15.21 mmol)^21^. The mixture was stirred at this temperature for 20 min and then cooled to −78 °C. A solution of aldehyde (7.93 mmol 1.73 g) in THF (21.7 mL) was slowly added and the reaction mixture and stirred at −78 °C for an additional 60 min. The mixture was allowed to warm to 0 °C. A saturated solution of ammonium chloride (15 mL) was added and the mixture extracted with EtOAc (3 × 20 mL). The organic layer was washed with water (2 × 10 mL) and brine (20 mL), dried over MgS0_4_, and concentrated to give 6.33 g red oil. The residue was purified on a short SilicaGel column (95:5 hexane: diethyl ether) to give 1.85 g (71% yield) of the desired ester. 9-*cis*-retinyl ester was separated from its *trans* isomer by preparative HPLC (cosmosil cholester packed column 20.0 mm I.D × 250 mm) using elution of CH_3_OH / H_2_O, 90:10 for 4 min then, 90:10 to 95:05 for 4 min, and then 95:05 for 17 min (flow rate 25 mL/min, λ = 402 nm). Retention time was 13.7 min. Analytic HPLC analysis was performed using cosmosil cholester packed column (4.6 mm I.D × 250 mm) using the following method: isocratic elution of CH_3_OH / H_2_O 95:05, in 12 min (flow rate 2 mL/min). Retention time was 8 min (99.6% purity). ^1^H-NMR (CDCl_3_, 400 MHz);7.08 (dd, 15, 11.3, 1 H), 6.65 (d, 16, 1 H), 6.29 (d, 15, 1 H), 6.22 (d, 15, 1 H), 6.05 (d, 11.9, 1 H), 5.77 (s, 1 H), 4.17 (q, 2 H), 2.34 (s, 3 H), 2.05 (t, 2 H), 2.00 (m, 3 H). 1.75 (s, 3 H), 1.64 (m, 2 H), 1.49 (m, 2 H), 1.28 (t, 3 H), 104 (s, 6 H). MS (ES + ) calculated for C_22_H_32_O_2_ was 328.5, found 329.4 (M-H + ). 2D COSY NMR experiment (700 MHz) proved that 9-*cis*-retinyl ester was obtained.

### Ester reduction (9-cis-retinol formation) (step e)

To a solution of ethyl 9-*cis*-retinyl ester (1.674 mmol 0.55 g,) in THF (5 mL) at −78 °C, DIBAL-H (7 mL, 1 M in hexane, 7 mmol) was added and the resulting suspension was stirred for 1 h at −78 °C^22^. After careful addition of 10% aqueous NH_4_Cl, the mixture was extracted with ethyl ether (3 × 10 mL). The combined organic layers were dried over MgSO_4_ and concentrated using rotary evaporator. The residue was used in the next step without further purification. Analytic HPLC analysis was done using cosmosil cholester packed column (4.6 mm I.D × 250 mm) and the following method: Elution of CH_3_CN/H_2_O, 65:35 to 80:20, in 20 min, and then 100% CH_3_CN for 8 min (flow rate 2 mL/min, λ = 370 nm). ^1^H-NMR (400 MHz) showed typical peaks of the alcohol group at 4.25 ppm (2 H) and 3.71 ppm (OH, broad peak). Retention time was 13.4 min (87% purity). MS (ES+) calculated for C_20_H_30_O was 286.45, found 269.3(M-(OH)).

### 9-cis-retinyl phosphonium salt formation (step f)

To a solution of 9-*cis*-retinol (0.77 mmol, 220 mg,) in dry MeOH (1 ml), triphenylphosphine hydrobromide (0.88 mmol, 320 mg in MeOH) was added dropwise with stirring. The mixture was stirred at room temperature under argon. After 1 h, the solvent was evaporated in a rotary evaporator and the light orange residue was washed with 10 ml portions of hexane 5–6 times to remove side products. Analytic HPLC analysis (cosmosil cholester packed column 4.6 mm I.D × 250 mm) was performed by elution of CH_3_CN (0.1% TFA) / H_2_O (0.1% TFA), 65:35 for 4 min, then from 65:35 to 75:25 for 8 min, and finally isocratic elution 75:25 CH_3_CN (0.1% TFA) / H_2_O (0.1% TFA) for 4 min (flow rate 2 mL/min, λ = 370 nm). Retention time was 10.8 min (72% purity). MS (ES+) calculated for C_38_H_44_P+ was 531.3, found 531.4 (M), 532.3 (M-H+ ).

### Wittig reaction 9CBC formation (step g)

**Figure 3:**
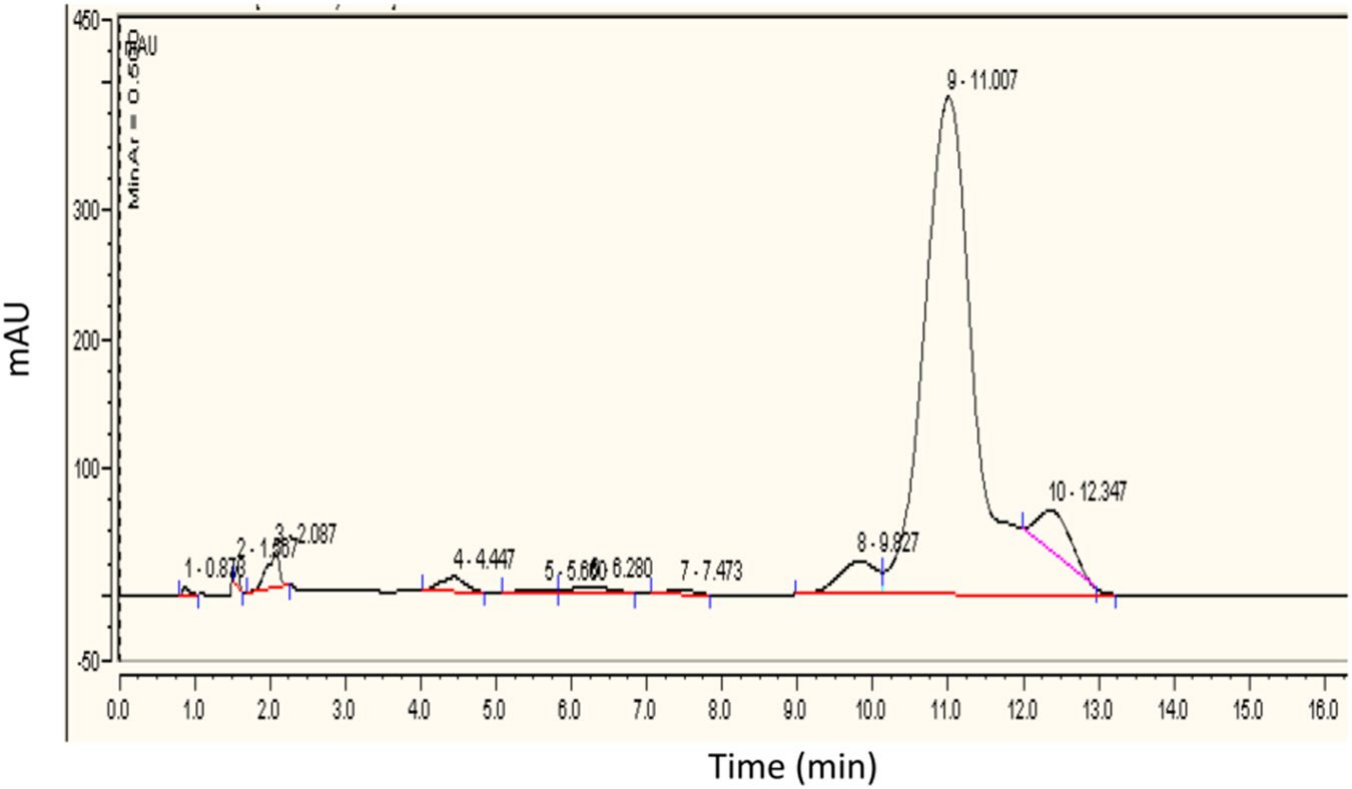
HPLC reversed phase analysis of 9CBC reaction mixture. (λ = 447 nm).

**Figure 4:**
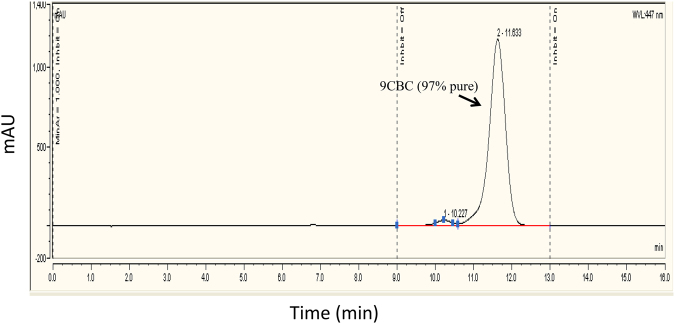
HPLC analysis (λ = 447 nm) of purified 9CBC (8:2 CH_3_OH:CHCl_3_).

To a stirred solution of 86 mg of 9-*cis*-retinyl triphenylphosphonium bromide (0.14 mmol) in 2 ml of dry ethanol at 70 °C, all-*trans* retinal (19.7 mg, 0.069 mmol) in 17 ml of dry ethanol was added dropwise^19^. KOH (0.43 g) dissolved in dry ethanol (5 ml) was added slowly to the mixture, and the solution was stirred at 70 °C for 15 min and then at room temperature for 2 h. The reaction was monitored by TLC (8:2 hexane:ethyl acetate). When no more carotene formation was noted after 2 h, water (10 ml) was added to the reaction mixture and the product was extracted with portions of diethyl ether. The organic phase was evaporated under vacuum. MS calculated for C_40_H_56_ was 536.4, found 536.4 (M) and 537.4 (M-H+). HPLC analysis of the crude (cosmosil cholester packed column 4.6 mm I.D × 250 mm) isocratic elution of CH_3_OH / CHCl_3_, 80:20 for 16 min (flow rate 2 mL/min, λ = 447 nm). Retention time was 11 min, (87% purity, Fig. 3). 9CBC was purified by preparative HPLC (Cholester packed column) using elution of CH_3_OH:CHCl_3_, 85:15 for 5 min, then 85:15 to 80:20 for 5 min and then isocratic elution of 80:20 for 20 min (flow rate 25 mL/min, λ = 447 nm). Retention time was 16 min. MS calculated for C_40_H_56_ 536.4, found 536.4 (M) and 537.4 (M-H+). Further purification using preparative HPLC resulted in 9CBC at 97% purity. Figure 4 demonstrates the analytic chromatogram of the main fraction obtained (λ = 447 nm) using isocratic elution of 80:20 CH_3_OH:CHCl_3_.

It is also possible to substitute CHCl_3_ with methylene chloride for preventing possible damage during separation. Likewise, the final separation can be done with methyl-*tert*-butyl ether and acetonitrile, instead of the solvents mentioned above.

### Stable formulations of 9-cis-β-carotene

Purified 9CBC was found to be unstable under vacuum or nitrogen flushing. In this study, various 9CBC formulations involving a natural or synthetic commercially available antioxidant(s), a viscosity enhancing agent, or their mixture, were prepared, and the chemical stability of some of these formulations was tested. The specific formulations prepared were as following:

1. Lecithin + antioxidants mix [a pellet content from the commercially available product CarotenALL, Mixed Carotenoid Complex (Jarrow Formulas)]+9CBC (10:5:1 w/w/w)
2. Lecithin + BHT + 9CBC (10:5:1 w/w/w)
3. Miglyol 810 + antioxidant mix + 9CBC (100:5:1)
4. Miglyol 810 + BHT + 9CBC (100:5:1)
5. Tween 40 + 9CBC
6. Tween 20 + antioxidant mix + 9CBC
7. Tween 40 + antioxidant mix + 9CBC

Table 1 shows spectral tests confirming the stability of 9CBC in the formulations tested within the specified time period. As clearly indicated, lecithin as an excipient and binder plays a crucial role in stabilizing 9CBC. Interestingly, although a formulation comprising an antioxidant only as the anti-degradative measure has somewhat of a positive effect during the first 14 days, the formulation has rapidly degraded during the next days, wherein the measurable 9CBC by day 22 was almost as low as in the blank formulation. In fact, as shown in Table 1, the amount of 9CBC in an antioxidant-based formulation was significantly lower than that in the formulation that further contained a viscosity enhancing agent (lecithin), already after 14 days, suggesting that 9CBC degradation starts during the first 14 days and intensifies later. In sharp contrast, the measurable amount of 9CBC in the formulation containing both antioxidant and a viscosity enhancing agent was almost 100% compared to the initial amount, indicating the stability of formulation.

**Table 1:** Results of spectral tests of the various formulations tested.

Tested medium	Absorption at 447/450 nm 14 days	Absorption at 447/450 nm 22 days
Diethyl ether	0	0
Diethyl ether+BHT+9CBC	2.11/2.08	1.05/0.96
Diethyl ether+lecithin+BHT+9CBC	2.43/2.32	2.42/2.30

Demonstrating the preservative effects of the various formulations tested on the 9CBC.

### Examination of the toxicity of vehicles of synthetic 9CBC in cultures of RPE65rd12 mouse eye cups

To examine the biological activity of the synthetic 9CBC we chose a mouse model of retinoid cycle defect, RPE65rd12^23^. The RPE65rd12 mice carry a null mutation in the gene encoding the retinoid cycle RPE65 isomerohydrolase and are a widely-used model for *RPE65* deficiency. RPE65 is essential for regenerating the rod and cone chromophore 11-cis retinal after it is bleached during light absorption^24^. Loss of RPE65 function results in death of photoreceptors and causes Leber congenital amaurosis (LCA), a severe form of RP^25^. We have recently shown that feeding these mice with an extract of *Dunaliella Bardawil* algae rich in 9CBC rescued retinal function and preserved cone structure^26^, suggesting that this model may appropriate for testing the biological activity of the synthetic 9CBC. Since only small amount of synthetic 9CBC was available, we examined the biological activity of 9CBC *in vitro* in eye cup cultures of these mice. Since the BCMO1 enzyme is expressed in the retina in the RPE cells^12^ we chose to use whole eye cups that maintain the neuro-retina attached to the underlying RPE tissue.

First, we examined the toxicity of different vehicles to determine which vehicles are compatible for these *in vitro* experiments. To this aim, eye cups from RPE65rd12 mice at age of 21days were cultivated *in vitro* for 18 hours in the presence of different vehicles presented in Table 2. Toxicity of compounds was examined following staining for cell death (apoptosis) by TUNEL assay and scored by two masked observers as indicated in the “Methods” section. As shown in Table 2, addition of Miglyol 810 for enhanced viscosity and the “antioxidant mix” at a volume equivalent to addition of 1 μM 9CBC resulted in minimal apoptosis in the neuroretina in these cultures. Based on these findings it was decided to progress with Step f (Fig. 1) – 9-cis-retinyl phosphonium salt formation and use Miglyol 810+ “antioxidant mix” as carriers for 9CBC as these reagents demonstrated low toxicity *in vitro*.

**Table 2:** Toxicity of vehicles in RPE65/rd12 mouse eye cup cultures.

Vehicle*	Concentration	Apoptosis score	Number of eye cups tested
None (growth media)		0	14
Phosphonium salt	0.01 mM	1	2
Phosphonium Salt	0.1 mM	1	2
Ethanol	5%	1	6
BHT	Volume equivalent to addition of 10 μM 9CBC	4	4
Miglyol 810	Volume equivalent to addition of 10 μM 9CBC	1	2
“antioxidant mix”*	Volume equivalent to addition of 10 μM 9CBC	1	6
Tween 40 and “antioxidant mix”*	Volume equivalent to addition of 10 μM 9CBC	4	3
Miglyol 810 and “antioxidant mix”*	Volume equivalent to addition of 1 μM 9CBC	1	3
Miglyol 810 and “antioxidant mix”*	Volume equivalent to addition of 10 μM 9CBC	3	3

^*^“antioxidants mix” - a pellet content from the commercially available product CarotenALL, Mixed Carotenoid Complex (Jarrow Formulas) and 9CBC (10:5:1 w/w/w).

### Examination of the biological effects of synthetic 9CBC in eye cup cultures of RPE65rd12 mice

**Figure 5:**
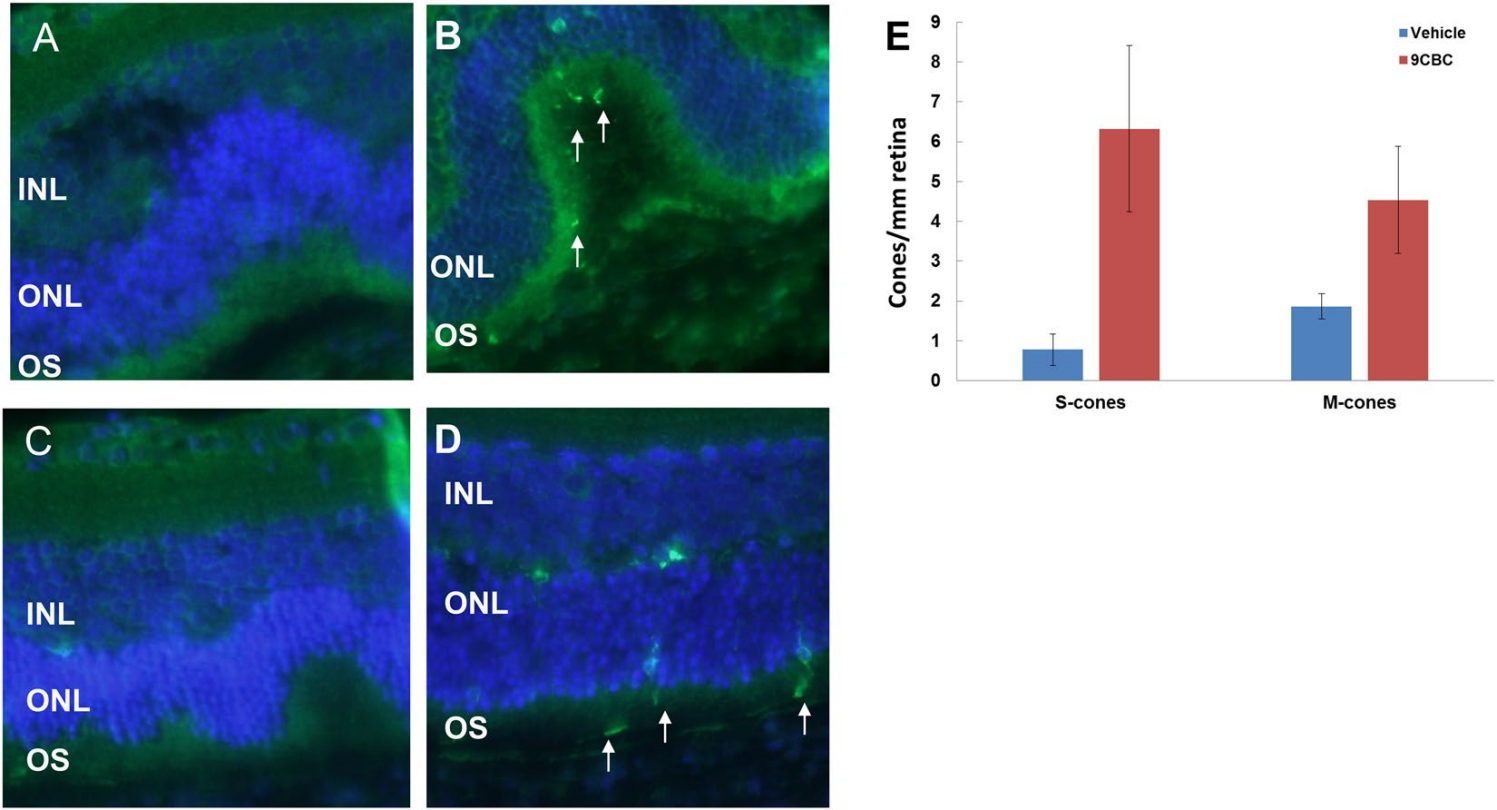
Synthetic 9CBC rescues S- and M-cones from degeneration in eye cup cultures of RPE65rd12 retina. Eye cups of 21 day old RPE65Rd12 mice were incubated in media supplemented with 1 μM synthetic 9CBC (4 eye cups, panels B & D) or same concentration of vehicle solution (3 eye cups, panels A & C) for 18 hours. Eye cups were fixed and sections were stained with antibodies directed against S-cone opsin **(**green, marked with white arrows, panel A, B) or M-cone opsin **(**green, marked with white arrows, panels C, D) and counter stained with DAPI (blue). (**E**) Number of positively stained cells per section was recorded and data are presented as mean ± standard error.

Next, we examined the biological activity of this formulation of the synthetic 9CBC in retinal eye cup cultures of RPE65rd12 mice. As shown in Fig. 5, in control cultures that contained the carriers Miglyol 810 and “antioxidant mix”, hardly any positive staining of s- and m-opsin was evident throughout the eye cups sections, suggesting that both S- and M-cones degenerated (Fig. 5A,C and E). By contrast, in eye cups incubated with 1μM synthetic 9CBC formulated with the Miglyol 810 and the“antioxidant mix”,over 2- and 9- fold higher m-opsin and s-opsin positive staining were observed, respectively, compared with control cultures [mean ± SE: 4.53 ± 1.34 vs. 1.83 ± 0.32 m-opsin/mm retina, p = 0.05; 6.33 ± 2.11 vs. 0.77 ± 0.39 s-opsin/mm retina, p = 0.034, respectively, Fig. 5B,D and E]

There was no significant difference in rhodopsin staining between control cultures and cultures incubated with 9CBC (data not shown).

## Discussion

In this study we demonstrated a practical synthetic route for biologically active 9CBC.

Synthetic 9CBC rescued both M- and S-cones from degeneration in cultures of RPE65rd12 mouse eye cups, increasing by 2- and 9- fold the number of cones in the retina, suggesting that the synthetic 9CBC compound may have a therapeutic effect in the degenerating retina. These findings are in accordance with the biological activity of the natural form of 9CBC. Thus, previous clinical trials demonstrated retinal function rescue by oral treatment with 9CBC rich *Dunaliella Bardawil* algae extract in patients with retinitis pigmentosa and defects in the retinoid cycle^13,14^. In addition *in vivo* animal studies suggested that feeding with 9CBC rich *Dunaliella Bardawil* algae extract rescued retinal function and M-cone cell structure *in vivo* in RPE65rd12 mice^26^ and 9CBC extracted from the algae rescued M-cone photoreceptors from degeneration in cultures of RPE65 knockout mice eyes^27^. Our findings that synthetic 9CBC had no significant effect on rhodopsin expression, are in agreement with the biological activity of the algae 9CBC in previous clinical studies and *in vivo* animal studies as well as with the results of Ozaki *et al*.^13,14,27^. In future studies we aim to upscale the production of the synthetic 9CBC molecule and test the therapeutic activity and safety of the synthetic molecule *in vivo* in the RPE65rd12 mouse model as a step towards clinical trials.

The motivation of the presented study was to develop the robust experimental protocol for synthetic preparation of 9CBC from inexpensive building materials. We have synthetized 9CBC by step-by-step method from 3,3-dimethylacrylate, but we also can use the alternative approach to start from a commercially available β-Ionone as a starting platform for preparation of the target 9CBC product. Thus, the main focus of next stage of this study is development of a practical cost-effective synthetic route to 9CBC, including the process optimization and further up-scaling leading both to the significant lowering of the 9CBC cost. Nevertheless the main obstacle in the cost effectiveness of 9CBC synthesis is preservation of the final product from degradation, that we successfully overcame using appropriate formulations.

9CBC intermediates were characterized by NMR, MS and HPLC. The final product was found extremely unstable under drying (evaporation of HPLC solvents) by both: vacuum or nitrogen flushing. Most examined formulations proved to be unstable during storage in the presence of light and air, with 9CBC degraded by photodegradative/oxidative etc. Several tests were performed in HPLC (cosmosil cholester packed column 4.6 mm I.D × 250 mm) in order to determine its stability conditions. A number of stabilizers were used in order to preserve 9CBC: butylated hydroxytoluene (BHT) and tert-butylhydroquinone (TBHQ) soybean oil, L-α-lysophosphatidylcholine from glycine max, lecithin, miglyol, tween-40, dimethyl sulfoxide (DMSO) etc. The efficacy of a range of synthetic and natural antioxidants in preventing or at least significantly minimizing degradation of 9CBC during HPLC purification, evaporation and storage were determined. The formulations were spectrally tested for active agent stability within time frame of 1 month.

Some of the antioxidant formulations were toxic in the eye cup cultures *in vitro*. However as these compounds are commonly used as food supplements (e,g. BHT) we plan in future studies to test these formulations *in vivo* in the RPE65rd12 mouse model where their toxicity is predicted to be significantly reduced.

In conclusion, this study presents a novel, convenient and economical synthetic route for biologically active 9-*cis*-β-carotene. Up scaling the production of 9CBC may pave the way to development of treatment for incurable retinal dystrophies and other diseases including psoriasis and atherogenesis.

## Methods

### Materials

Ethyl 3,3-dimethylacrylate was purchased from Sigma Aldrich. b-Cyclocitral was purchased from Alfa Aeser. All trans retinal and triethyl 3-Methyl-4-phosphono-2-butenoate were purchased from TRC. Triphenylphosphonium bromide was purchased from Acros Organics. alpha,alpha-Azoisobutyronitrile (AIBN) was purchased from Molekula. Soybean, Mw = 537.7 gr/mol, purchased from Sigma. All reactions were carried out under argon/nitrogen atmosphere. The reactions (except for steps a and b in Fig. 1) were performed in the dark under red dim light.

### Synthesis of 9-cis-β-carotene

A multi-steps process for the synthesis of 9CBC from common available compounds was developed based on stereospecific synthesis of 9-cis olefin intermediate via a lactone ring opening with complete retention of double bond configuration, followed by Horner-Emmons, ester reduction and Witting reaction to produce the final compound. The method for the synthesis of 9CBC included:

(i) reducing the lactone 4-methyl- 6-(2,6,6-trimethylcyclohex-1-en-1-yl)−5,6-dihydro-2H- pyran-2- one (3) and opening the ring of the lactol obtained (4) with complete retention of the double bond configuration, to obtain (2Z,4E)−3-methyl-5-(2,6,6-trimethyl cyclohex-1-en-1-yl)penta-2,4-dienal (5); (ii) subjecting the (2Z,4E)−3-methyl-5-(2,6,6-trimethylcyclohex-1-en-1-yl) penta-2,4-dienal to Horner-Emmons reaction to obtain a 9-cis retinyl ester (6); (iii) reducing the 9-*cis*-retinyl ester to obtain 9-*cis*retinol (7); and (iv) converting the 9-*cis*-retinol to its triphenylphosphonium salt (compound 8), and subjecting of the triphenylphosphonium salt to Wittig reaction with an aldehyde in the presence of a strong base, to obtain 9CBC. In a particular example, the triphenylphosphonium salt of 9-*cis*-retinol is subjected, in step (iv), to Wittig reaction with all-*trans* retinal to obtain 9CBC. The 4-methyl-6-(2,6,6-trimethylcyclohex-1-en-1-yl)−5,6-dihydro-2H-pyran-2-one (3) used as a starting material in the method may be synthesized by any suitable process.

### Animals

RPE65/rd12 mice were used in this study^23^. Mice were raised and bred at the Sheba Medical Center animal facility. All animal procedures and experiments were conducted with approval and under the supervision of the Institutional Animal Care Committee at the Sheba Medical Center, Tel-Hashomer, and conformed to recommendations of the Association for Research in Vision and Ophthalmology Statement for the Use of Animals in Ophthalmic and Vision Research.

### Eye cup cultures

Fifty two eyes were enucleated from 21 day old RPE65/rd12 mice, and the posterior segment was separated from the cornea, the lens, the iris and the ciliary body under a surgical microscope. The eye cups were placed onto a microporous membrane (30 mm in diameter; Millicell-CM; Millipore, Bedford, MA) with the ganglion cell layer (GCL) facing up and the sclera facing the filter in a six-well culture plate. Each well contained 1 ml of culture medium consisting of 50% minimum essential medium/HEPES (Sigma, St. Louis, MO), 25% HBSS (Invitrogen, USA), and 25% heat inactivated horse serum (Invitrogen, USA) supplemented with 200 μM L-glutamine and 5.75 mg/ml glucose and 1μM synthetic 9CBC or vehicle as indicated in the text. The eye cups were maintained in culture at 37 °C, 5% CO_2_ incubator for 18 hours.

### Histology analysis

Tissues were fixed in formalin and embedded in paraffin as previously described^28^. Cross sections (4 µm) were cut along the vertical meridian of the eye through the optic nerve and were stained with hematoxylin and eosin. TUNEL staining was performed following manufacturer instructions (Roche, Germany). For detection of rhodopsin and cone opsins, paraffin sections were incubated following citrate buffer antigen retrieval with anti-rhodopsin antibody (RET-P1, Abcam), anti S- (Millipore) and M- (Millipore) opsin and Alexa Fluor® 488-AffiniPure Donkey Anti-Mouse IgG antibody (Jackson Immuno Research). Following counterstaining with DAPI, sections were examined and photographed by a fluorescent microscope (Olympus BX51).

The Kruskal-Wallis test was used for comparison of m- and s-opsin staining in 9CBC treated vs. control eye cups. Statistical analysis was performed using SPSS for windows version 20.0 (IBM).

### Scoring of apoptosis rate

To score the apoptosis rate in the eye cups, sections of eye cups were stained as indicated above and examined by two masked observers. Sections were assigned a score on the scale of 0–4: 0 – no cell death in neuro-retina, 1 – less than 10% of photoreceptors are TUNEL positive, 2–10–50% of photoreceptors are TUNEL positive, 3 – more than 50% of photoreceptors are TUNEL positive, 4- massive cell death in all neuro-retinal layers.
